# Partnering with persons in long-term recovery from substance use disorder: experiences from a collaborative research project

**DOI:** 10.1186/s12954-019-0310-x

**Published:** 2019-06-24

**Authors:** Henning Pettersen, Morten Brodahl, Jeanette Rundgren, Larry Davidson, Ingrid Amalia Havnes

**Affiliations:** 1 Norwegian National Advisory Unit on Concurrent Substance Abuse and Mental Health Disorders, Mental Health Division, Innlandet Hospital Trust, P.O. Box 104, N-2381 Brumunddal, Norway; 2 Inland Norway University of Applied Sciences, P.O. Box 400, 2418 Elverum, Norway; 3 Program for Recovery and Community Health, School of Medicine, Yale University, Erector Square 319 Peck Street, New Haven, CT 06513 USA; 4 Norwegian National Advisory Unit on Substance Use Disorder Treatment, Oslo University Hospital, P.O. Box 4959 Nydalen, 0424 Oslo, Norway

**Keywords:** Methodological study, Collaborative research, Substance use disorder, Recovery

## Abstract

**Background:**

Traditional research about substance use disorder (SUD) treatment is considered, among an increasing number of service users, to be disempowering and poorly reflective of their priorities. Thus, this methodological article sought to examine the experiences of a peer research group (PRG), whose four members were in long-term SUD recovery, and a principal investigator (PI), when collaborating on a study of SUD recovery. This article has also aspired to discern the influence of peer researcher participation on the research process. The purpose of the qualitative research project that formed the basis of this methodological study was to examine the reasons provided and strategies employed for abstaining from problematic substance use among persons with SUDs.

**Methods:**

The project took place from 2015 to 2018, during which time individual interviews were conducted with 18 persons in recovery from SUDs. The PRG contributed to all parts of the project and worked alongside the PI in preparing the study, during early stages of data analysis, and while writing up the findings. In total, ten group discussions were held over the course of 3 years.

**Results:**

The study showed that the PRG offered important contributions with respect to developing the interview guide, preunderstanding among the PRG members, and discussing alternative forms of data collection. Key findings about how this collaborative research process was experienced relate to three matters: the group aspect of participation, the value of predictable routines and clear expectations, and the open sharing of private matters. The PI experienced the research process as having been enriched by alternative ways of asking questions and interpreting findings and as an interactive arena for reciprocal social and professional support.

**Conclusions:**

When establishing a PRG while studying recovery processes, it can be advantageous to include several peer researchers with diverse lived experiences concerning substance use, treatment, and recovery. If possible, at least one peer researcher with formal training or qualitative research experience might be included. The PI should be trained in collaborating with peer researchers or should be part of a research environment in which it is possible to discuss methodological challenges with other researchers.

## Background

Traditional research about mental health and substance use disorder (SUD) treatment is considered, among an increasing number of service users, to be disempowering and poorly reflective of their priorities [1, 2]. Persons with user experiences see the need to bring more depth and detail into the knowledge production, and often wish to participate in the initial phases of a project in order to contribute to more precise aims and research questions [3]. Further, collaborative research, in which service users are included in the research process, is valuable for bringing different perspectives at all levels of accountability into the process. This inclusion is a quality assurance safeguard that generates important questions from different vantage points, which are highly relevant to all levels of health and care services, and which help to improve the evidence base that informs how health care services are both designed and provided [4, 5].

Collaborative research that builds upon the direct experiences of the co-researchers with user experiences during the data analysis and writing phases has rarely been undertaken in previous SUD research. Such an approach has the potential to increase the quality, relevance, and utility of the findings being generated [6, 7]. Furthermore, collaborative research about SUD recovery holds potential for capacity building and empowerment among both service users and the affected communities, as has been indicated in the national policy documents [8, 9] and evidenced in research [10–13]. Thus, this methodological article sought to examine the experiences of both peer researchers and a principal investigator who collaborated on a study of SUD recovery and to examine how peer researcher participation influenced the research process.

## Methods

This article describes the experiences of individuals who participated in a collaborative research project about the topic of SUD recovery. As such, it has entailed a naturalistic form inquiry [14], in which we have employed an exploratory and descriptive approach [15, 16]. With respect to the study of SUD recovery for which the research process described in this article was undertaken, two scientific articles [17, 18] and one popular scientific article [19] have been published; one scientific article is in press [20]. This article provides a description of the research process (see Table 1).

**Table 1:** Overview of the research process

Time frame	2015		2016	2017–2018		
Process	Preparation	Group discussions 1–4	Group discussions 5–9	Articles published/in review	Group discussion 10	Principal investigator
Method and objective	Establishing agreement to include patients from two cohort studies (COMORB)	Establishing the PRG. Agreement on working conditions. Feedback on and review of the interview guide	Data analysis of the working steps and of the PRG members’ experience of the process	Journal publications and dissemination. PRG members as co-authors and dissemination to remaining PRG members	Data analysis of the PRG members’ experience of the process	Reflections on the research process with the PRG
Data material (reports, recordings, transcribed interviews)	Reports from three meetings. Obtaining approval from the Ethical Committee for Health Research Ethics	Reports from four meetings	Reports, digital recordings, and transcriptions from five meetings.	Art. (1) Why those with SUD stop abusing substances? Art. (2) Helpful ingredients in the treatment of SUD (in press) Art. (3) How social relationships influence SUD recovery Art. (4) A popular scientific article based on article 1, published in Norwegian Each article distributed to the PRG	Report, digital recording, and transcription of a 2-h meeting	Summary of continuous notetaking

### Context

The overall purpose of the qualitative research project that forms the basis of this methodological study was to examine the reasons provided and strategies employed for abstaining from problematic substance use among persons with long-term SUDs. The project took place from 2015 to 2018 [17, 18, 20]. Participants were recruited from the Comorbidity Study: Substance Dependence and Co-occurrent Mental and Somatic Disorders (COMORB study). The COMORB study is a longitudinal study of two cohorts from Norway concerning mental [21, 22] and somatic [23] comorbidity, respectively. The two cohort studies are (1) an 18-year follow-up of a dual diagnosis study on psychiatric comorbidity in a heterogeneous sample of patients with SUDs and (2) a 20-year follow-up of a study on opioid maintenance treatment, for which somatic morbidity among dependent opioid users was assessed before, during, and after treatment. These two cohorts were merged for joint data collection in 2015 (*N* = 148). The qualitative research project recruited participants from this joint cohort in 2016.

Semi-structured interviews were undertaken by the principal investigator (PI) among 18 persons with a SUD who had been abstinent for at least 5 years. All interviews were transcribed verbatim, and a combination of systematic text condensation [24] and narrative analysis [25, 26] was applied in working with the interview data. A group of four peer consultants with experiences of long-term recovery from SUDs contributed to all parts of the project and worked alongside the PI in preparing the study, during early data analysis stages, and while writing up the findings.

### Establishing the peer research group

During the process of recruiting the peer research group (PRG), three meetings were arranged to provide general information about the research project and the conditions that should guide the PRG involvement. Two PRG members were included from the start. The other two were recruited following suggestions from the first ones, and they joined the group during the second meeting. The PI and the two initial PRG members were colleagues in the Norwegian National Advisory Unit on Concurrent Substance Abuse and Mental Health Disorders (NROP) and enjoyed a positive collegiate relationship, though without a prior history of collaborative research. The two initial PRG members thought it important to also include persons from other organizations and with experiences from different types of SUD treatment. Since the two initial PRG members had a substantial network of persons in recovery from SUDs, it was convenient to recruit more PRG members from that network. This recruitment strategy was agreed upon by both the PI and the two initial PRG members.

Initially, we established written agreements with each member of the PRG concerning (1) non-disclosure of confidential information; (2) general information about the project, including the meeting location and other practicalities; and (3) payment (NOK 450/h).

The meeting attendance rate among the PRG members was high; two of the PRG members attended all nine meetings, one attended eight, and one attended six.

### Members of the peer research group

Four persons who all had experienced both SUD and stable recovery for 3–10 years were recruited to the PRG. “Stable recovery” encompasses both total abstinence from all psychoactive substances and non-problematic use of legal substances. They had previously used a diversity of substances, quite similar to the study participants. Three were male, one was female, and they were of similar age. One of the PRG members had received 12 months of training in collaborative research, while the others had no formal research training, but had experience from operating or actively engaging in organizations for people with former or present substance use problems. Primarily, the PRG members were persons with first-hand knowledge of the studied phenomena: reasons and strategies for abstaining from problematic substance use.

### Planning and conducting the collaborative group meetings

**Fig. 1:**
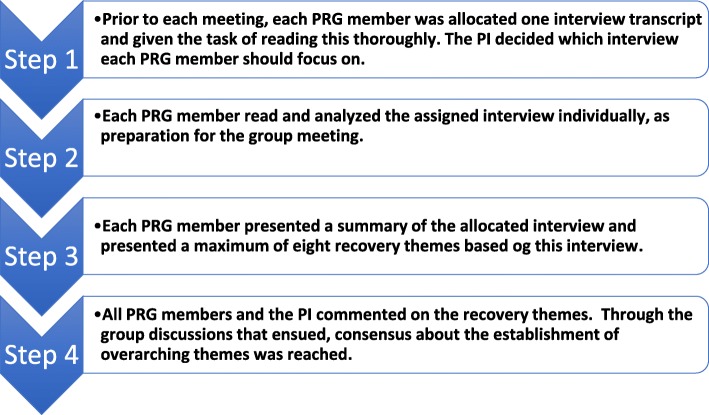
The working process for reviewing/analyzing interview transcripts as repeated every 4–5 interviews

The PI organized a total of nine meetings with the PRG throughout the research process (see Fig. 1). The PI sent each PRG member a reminder email 4–5 days prior to the meetings, together with a meeting agenda. The meeting discussions were led by the PI. Each of the nine meetings lasted 1–2 h, and the last five were digitally recorded. During each meeting, notes were taken by the PI and then e-mailed to the members of the PRG the following day.

The first four meetings (August 2015–February 2016) involved establishing agreement about project documents; reviewing the overall study purpose, research questions, inclusion criteria, and interview guide; and reaching consensus about central concepts. During the same period, the PI reviewed and recruited eligible participants from the overall COMORB study and made agreements for conducting individual interviews with those who both met inclusion criteria and consented. The subsequent five meetings with the PRG took place as these interviews were underway (March–September 2016). They involved group discussions concerning interview content, initial thematic developments, and the research process. As such, these subsequent five meetings are conceived of as having constituted an important, preliminary phase of systematic analysis for the project at large.

The PI conducted individual interviews with a total of 18 research participants. These interviews were conducted in their homes, and they were digitally recorded and transcribed verbatim. After each 4–5 individual interviews, each member of the PRG received one transcribed interview for individual reading. The purpose was to identify recovery themes throughout the text. During the fifth through the ninth meetings, the PI and the PRG discussed the themes that had been identified in each interview. Each member of the group read aloud a summary from the assigned interview, along with 4–8 recovery themes that this member had identified. At the end of this presentation, the rest of the group had the opportunity to comment on the identified themes. Finally, when all 4–5 interviews had been presented and commented upon, the group discussed and reached a consensus about eight themes that were understood as relevant for and representative of the interviews. This procedure was undertaken four times, once in each of the fifth, sixth, seventh, and ninth meetings between the PI and the PRG, in order to cover all 18 interviews.

During the eighth meeting, a review of the overall themes that had been established during the prior group discussions was also undertaken. The PRG members suggested adapting the interview guide so as to include additional topics in subsequent interviews. These suggestions were based on findings from the initial analyses and the PRG members’ own SUD and recovery experiences. As a result, after having completed 14 of the 18 interviews, the interview guide was adjusted accordingly.

The ninth meeting was held for the main purpose of reviewing the last four interview transcripts and establishing the recovery themes that emerged from these, as had been the case with the previous meetings and transcripts, but these themes were then also compared to the themes that had been established for interviews 1–14. During all nine meetings between the PI and PRG, members of the PRG also provided feedback, including critical comments, about the research process.

The next stage of this process of analysis involved the PI emailing each PRG member the transcripts from the last five meetings, which had been audio recorded. This provided the PRG members with the opportunity to examine their own comments and arguments during the discussions, to provide feedback to the PI regarding any passages that they perceived as incorrectly transcribed, and to provide further comments on the issues that had been brought up during the meetings.

One member of the PRG (MB) participated further in the research process by working together closely with the PI during subsequent analysis and while writing up the three research articles. Part of this work was carried out in collaboration with a research group (both peers with SUD experience and professional researchers without) at the “Program for Recovery and Community Health” at Yale University, during 2017 and 2018. One matter that was raised and emphasized by the PRG was the importance of disseminating the results of the project in everyday language and in non-scientific environments. Suggestions from the PRG were to arrange community meetings, write newspaper articles, spread information through social media, and bring forward the findings during regular meetings in the user organizations that each of them represented. One of the peer researchers also suggested writing a popular scientific article in order to communicate our findings outside of the scientific environment. Thus, besides submitting to and publishing in traditional peer-reviewed journals [17, 18, 20], the first article was also published as a popular scientific article in a Danish journal that is intended for treatment providers and the general public, including individuals with previous or present SUDs and their next of kin [19].

Finally, a tenth meeting was held in December 2018; two of the PRG members participated in this meeting along with IAH and the PI. The aim of this discussion was to focus on how the PRG members and the PI had experienced the research process. IAH was invited to participate in the meeting because she had experience from several qualitative research projects that did and did not include peer researchers and because she had not participated in the project at the former stages. Thus, she could launch questions about the research process from an external point of view.

### Analysis of the research process

An inductive form of thematic analysis [27] was engaged while examining the transcripts from both the group discussions held during the first year of the project and those from the group discussions held at the end of the project, two years later. An inductive approach entails identifying themes and codes that are strongly linked to and emergent within the data itself [28].

**Fig. 2:**
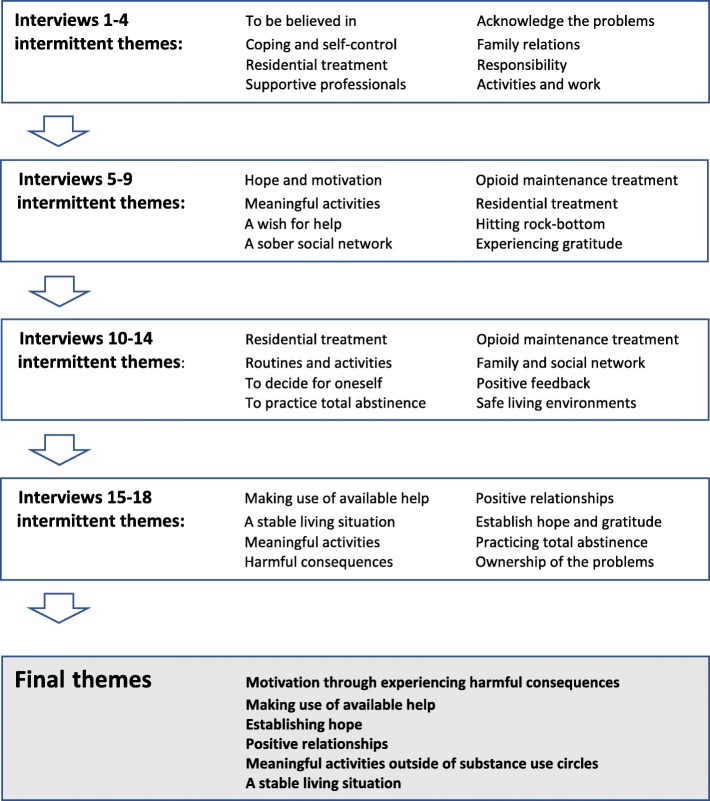
Theme development

The analysis moved through a six-stage procedure aiming to identify the shared themes across the group discussions (see Fig. 2). First, the transcripts were read and re-read systematically to acquire an overview of the data. Second, on the next reading, we searched the transcripts for sentences or paragraphs relating to the peer researchers’ experiences of the research process. During this procedure of identifying text relating to the research process itself, relevant text was marked with a code in the margins of each transcript. Third, similar codes were grouped together and compared with each other. Fourth, some of the initial codes became subthemes of a broader theme, and some codes were not used because they did not resonate with others. The codes were then organized into subthemes, followed by broader themes grounded in the subthemes. Fifth, the established themes were then compared to the initially coded text, to ensure that we did not exclude important parts. Based on the text underpinning each theme, we developed a thematic narrative for each. Then, based on discussions among the authors (HP, IAH, JR, and MB), we established suitable labels for each theme. The sixth and final step involved inserting relevant quotes from the transcripts into the text that matched the study aim and that illuminated or exemplified the contents of each theme.

The initial coding was performed by HP and IAH, but JR and MB contributed significantly throughout the later analysis and thematic development and in reading and revising all drafts of the manuscript. IAH contributed to the study from the unique standpoint of having not participated in the former qualitative study. Thus, she offered a unique perspective on the data material that was not informed by the preunderstanding of the research process found among the PRG members and the PI.

### Ethical considerations

The COMORB study was approved by the Norwegian Regional Committee for Medical and Health Research Ethics, South-East Region (REK-no. 2014/1936). When recruited for the present study, each member of the PRG signed a confidentiality agreement document along with a document confirming the duration of the project, frequency of the meetings, payment for meeting attendance, and expectations regarding individual preparations prior to meetings. The PRG members received payment according to the hours spent on meeting attendance and homework. Hours spent on homework varied among the PRG members, but they were paid for every hour spent on homework.

One important aspect of the study was to ensure anonymity for both the research participants and the members of the PRG. This implied that the peer researchers did not have any personal information about the research participants. Likewise, the research participants were informed that a group of persons with SUD and recovery experiences would be collaborating with the PI on the data analyses, but they were not aware of who these peer researchers were.

Furthermore, since the PRG members resided in the same geographical area as the study participants, deliberate precautions were taken to avoid allocating interview transcripts from acquaintances or persons who the PRG members were potentially familiar with. In addition to the previously mentioned confidentiality agreement that each of the PRG members signed, the PRG members and the PI agreed to avoid sharing any private experiences that were brought up during the group discussions with anyone outside the group. The PRG members also agreed to the digital recording and verbatim transcription of the group discussions, and the possible use of this data in a forthcoming study.

We did not establish a preemptive strategy for supporting peer researchers in the case of a worsening life situation or relapse during the course of the current project. In the last group meeting, we discussed the topic of whether or not a potential relapse would render one unfit to continue as a peer researcher in the project. To our knowledge, no one did relapse during the course of this project, so this was a hypothetical, but nevertheless significant discussion:

A: We have had some discussions about whether those who relapse shall be allowed to express themselves. But I believe that individuals still actively using substances also should be listened to. And I strongly believe that if one of us peers in this project had relapsed, then I cannot imagine us agreeing upon expelling that person from the group.

B: I agree, but people in general see this differently. Especially concerning having a clarified condition about our own illness. What this “clarified condition” really means, and how it influences our capacity to participate in research projects I really don’t know. A relapse does not necessarily signify a huge step backwards in life.

A: I was thinking … if one person participating in the project faces serious challenges, not necessarily substance use problems, then this person can still contribute with additional perspectives by having the experiences here and now. Such an attitude can also provide some growth and development.

In this quotation, the PRG members have raised the question of what a “clarified condition” implies. A “clarified condition,” as translated from Norwegian to English, refers to a clarified relation to one’s own illness and a sustained condition of recovery, in which illness symptoms do not interfere negatively with functional capacity for work and throughout everyday life. This is among the widely used, though not formalized, criteria for a professional role as a peer consultant with user experience in Norway. It was, in this project, a precondition for participation in the PRG. As we will return to later in this article, we suggest that future projects account in advance for the question raised by this PRG member and its implications for the ethical integrity of the study design. This is particularly important with respect to having a clear position on whether or not a relapse would disqualify one from further participation in the research process and with respect to a preemptive plan for whether or not relapse support would be offered and, if so, how.

## Results

Through notes taken from the initial four meetings between the PI and PRG, and through digital recordings and transcriptions from the final five meetings between the PI and PRG, as well as the tenth meeting that included IAH, we were able to analyze how the PRG influenced and contributed to the research process in collaboration with the PI and to describe the experiences of the research process as seen from the view of the PRG members and the PI.

### Collaborative activities

Our results about collaborative activities are based on data from the nine meetings that took place between the PI and the PRG. These activities concerned (1) developing the interview guide, (2) preunderstanding among the PRG members, and (3) discussing alternative forms of data collection.

#### Development of the interview guide

Prior to establishing the PRG, the PI had drafted an interview guide. This was presented to the PRG during the fourth group discussion.

One PRG member objected to several of the questions that had been included in the draft of the interview guide:

D: I am being confused by these questions. Half of them I wouldn’t be able to answer myself. The wording seems too academic to me, and that will probably be the case for the persons you are going to interview as well. I also think the interview guide has too many leading questions.

Critical feedback from the PRG predominantly concerned the use of words such as “strategies” and “rational” when asking “Which strategies have you employed to be able to quit using substances?” and “What is the significance of your own rational choices to be able to quit using substances?” Thus, “strategies” was substituted with “what have you done …,” and “rational” was simply omitted because it was superfluous and complicated the question.

A great amount of time during the group discussions that took place during meetings 4–9 was spent questioning the questions. This included both inquiring into the meaning of core concepts, such as recovery, abstinence, and treatment, as well as considering matters of open-ended versus leading questions and which questions should be included in the interview guide. The latter stages of the analytical, thematic development provided opportunities for the PRG members to engage their own lived experiences. When discussing how to prioritize each of the overarching themes and label them according to content, it became meaningful to illuminate different themes by drawing upon each PRG member’s experiences of SUD and recovery. Such themes related to, for example, diverse experiences of turning points and differing perspectives on whether respect is deserved and to be expected, or earned and to be gained, as well as questions of whether gratefulness is something experienced when happy or, the other way around, a catalyst for well-being and happiness.

The above discussions led to an adaptation of the interview guide, so as to include the following questions when conducting the last four interviews:

1) What was your way of thinking years back when you contemplated quitting, in comparison to your present thinking?

2) Is it possible to reach a turning point without having hit rock bottom?

3) What is the hallmark of a good service provider?

4) How does practicing openness or disclosure about your former substance use influence your recovery?

5) How can the impact of everyday events promote recovery?

#### Preunderstanding among the PRG members

All researchers bring a certain amount of preunderstanding into the research process. Reflexivity is an important aspect of qualitative methodology and analysis. The preunderstandings of the PI are discussed later in this article. In this section, we describe the preunderstandings among the PRG members and the ways in which these were explicitly recognized and managed throughout the analysis activities.

The initial phase of the analyses involved reading the interview transcripts with an open mind, so as to “bracket” the researchers’ interpretations [29]. A challenge for members of the PRG was to maintain a certain distance between their personal experiences and the topics brought up in these transcripts. For example, they found it difficult to descriptively summarize what each interview contained, without bringing in their own experiences from substance use and recovery. These challenges were not surprising, considering that the PRG members had intimate, first-hand experiences with SUDs, SUD recovery, and the topics under study and given that most of them had no formal research training. But, due to reminders from the PI, and often from PRG members themselves, about the importance of maintaining a certain personal distance while aiming to describe that which emerged from the data itself, this developed well.

As a way of illustrating how these discussions evolved, excerpts from different stages of the project are provided below. The first example relates to the analysis of an excerpt from an interview with one of the 18 study participants, concerning reasons for abstaining, which we previously presented and discussed in our first published article [17]:

A 48-year-old mother of two had used amphetamines for several years and had been abstinent for 17 years. She explained her reasons for abstaining:

The main reason to quit was in consideration of my two children. The oldest one lived with her father at that time, and the younger one I volunteered to place in a foster home. I thought it should be temporary, and it was really a wish of mine to keep a good relationship with both of them.

Important for participants was having their conscience weighing on them when they thought back to the troubled upbringing they had inflicted on their children. Several participants had their children taken and placed in foster homes by the child welfare system.

The following exemplifies the reflections among two of the PRG members during the sixth group discussion, when reviewing this interview.

D: I can actually recognize some of my own experiences in what she tells. Because I was in residential treatment in that period and it was time for my kids to visit me. Then I received a message from one service provider that my kids would not show up anymore. I was on the verge of leaving the treatment institution, because I found it very unfair. Shouldn’t I ever see them again? But then the treatment providers began talking about the importance of being able to take care of myself. If you cannot take care of yourself, you cannot look after your kids. That actually became the turning point for me, because then I was first priority. I really recognized a lot from my own life by going through this interview.

B: Yes, all of us recognize a lot when reading these interviews. But we have to focus on what the interviewed person tells, and not letting the ball roll too far. I admit having the same tendency myself, but we have to be cautious not to interpret too much, or ascribe the participants characteristics they don’t hold.

The final example illustrates how the reflective process about preunderstanding among the PRG members developed further in group discussion 10:

A: It was kind of challenging to read the interviews. I believe all of us became focused on comparing the interview contents with our own lives. Then we also had the tendency to convey our own experiences – and tell it out loud. You (B) have some education in collaborative research methods, in contrast to the rest of the group. Besides, you seem like a better listener than me. I am very much into talking. It was you that corrected us on keeping focus on what the interviews conveyed.

B: But we were supposed to focus on the information given in each interview, and not on ourselves. But we can reflect on why we did recognize ourselves in the interviews. Why do we recognize what we read and scrutinize? To me, it seems that I am unconsciously searching for data where I can mirror my own recovery process. Then I am not the only one with such experiences. Because it’s a lonely battle.

The examples above indicate that the PRG members had a tendency to interpret themes and findings in the interviews in light of their own experiences, at the expense of an impartial description of the data. They felt sympathy for the interviewees and found it challenging to be neutral. This was in part true for the PI also, considering his experiences of clinical practice and research. Through the intersubjective processes of the group discussions, it was possible to account for and manage this in ways that enabled greater focus on the descriptive aspect of the analysis.

#### Discussing alternative forms of data collection

The discussion of alternative forms of data collection involved a consideration of the possibility of employing focus group discussions in addition to personal interviews. The rationale for this suggestion, which was launched by one PRG member, was to explore, from a different vantage point, the additional aspects of the participants’ experiences that might emerge in a group discussion of substance use and recovery experiences.

One of the PRG members inquired:

B: Wouldn’t it be possible to conduct one or two focus group interviews with some of the participants? In particular, it would be interesting to see how they comment on each other’s experiences. That would be a great complementary to the individual interviews. Also, I could probably conduct some individual interviews myself. I believe we can come up with interesting results if they were interviewed by a user. I could do it if I was provided some training.

Unfortunately, the idea of conducting focus group interviews met challenges, because several of the 18 participants were skeptical about meeting and sharing experiences with other participants who they did not know. Thus, it was decided to proceed with the individual interviews only. Another discussion, as raised in the quotation just cited, was about whether one of the PRG members might conduct individual interviews. The argument was that being interviewed by a peer could elicit richer and potentially more truthful data/information, in the sense that the interviewees might, to a greater extent, open up and speak freely. The challenge was that the only member of the PRG who had some training in collaborative research (MB) did not have any training in interviewing. There was a possibility for him to receive training from a national peer-based competency center that utilizes the “user asks user” methodology, but this could not be realized within the time frame of the project. Thus, the PI conducted all of the individual interviews, followed by discussions of the interview transcripts in the meetings with the PRG.

### Experiences of the research process

Findings about the experiences of the research process and the PI’s reflections on it emerge predominantly from the data generated in the tenth meeting, in which these matters were explicitly reflected upon during the group discussion, but also, to a lesser degree, from the data from group discussions 1–9. These experiences concerned (1) the group aspect of participation, (2) the value of predictable routines and clear expectations, and (3) opening up about private matters.

#### The group aspect of participation

The PRG members frequently mentioned and commented on the advantages of participating together with other peers in a group, as opposed to being the only person in a research project with SUD experience. Three of the PRG members had never before participated in a research project of any kind, and they explained that their initial feelings of incompetency were alleviated by their membership in a group of peers.

As expressed by one PRG member:

A: Our privilege by taking part in this project is that we are four persons with user experiences meeting regularly. As far as I know, the most common in research projects is to recruit only one such person. The group aspect has been a great advantage for us in this project. It has been important that I have been listened to and experienced some kind of equivalence and reciprocity.

Additionally, the PRG members emphasized the importance of the composition of the group. Both genders were represented, they had diverse experiences of substance use, they had received different kinds of treatments, and they had been living quite different lives. They conceived of this heterogeneity as an advantage, and they regarded the group dynamic and positive energy during the discussions as stimulated by the differences among them. Furthermore, they felt that their interpretations of the transcribed interviews resonated, and they were able to focus on what they together agreed were the most important aspects.

#### The value of predictable routines and clear expectations

The PRG members described a common experience of unease in the early project phases, with respect to their lack of formal education and research training. They were recruited on the basis of their unique personal experiences with SUDs and SUD recovery, and they were not certain of what to expect from the PI or the project as a whole. Few of the PRG members were familiar with the reading of large documents or with the interpretative task of drawing the essence out of these texts. Because of this, they described it as helpful to have been given limited tasks that they could handle. As explained by one PRG member:

A: For most of us I believe it advantageous to have had only part of the material to go through. Some of us found it overwhelming to receive a lot of sheets filled with text. Indeed, we are persons with varying education and different experiences with the written word*.*

Another PRG member followed up, saying:

B: Yes, it has been a challenge for me as well. But at the same time, I am the kind of person who enjoys forms and statistics and such matters. That helped me a lot. For instance, when I went through the interviews doing colored coding, I became totally absorbed in the text and worked for several hours overtime because I found it so interesting.

Another matter of focus in the group discussions about experiences of the research process was related to how participating in a research project provided insights and therapeutic gain. By reading the narratives and exploring the experiences of other people while searching for recovery themes, the PRG members described having acquired a new perspective on the struggles in their own lives. Also, being able to manage the tasks allocated throughout the project gave the PRG members a strengthened sense of self-efficacy, particularly as the project progressed. The PRG members found it reassuring to participate in the group discussions, and they attributed their sense of security, in large part, to the structure and predictability of the clear expectations and group routines. Among the matters that they described having experienced as reassuring were the fixed structure concerning when and where to meet and the clear agreements about the work process and what was expected of each PRG member and of the collaboration between the PRG and the PI.

#### Opening up about private matters

All of the PRG members had been actively engaged in user organizations prior to participating in the current project. Thus, it was familiar and common for them to share their SUD experiences. The assurance of reciprocal anonymity between them and the participants of the larger study, and the confidentiality agreement within the PRG itself and with respect to the private matters that arose in these meetings, also seemed to facilitate openness among the PRG members. This can be contrasted with the attitudes of the 18 research participants who were interviewed. These research participants expressed discomfort with the idea of partaking in focus group interviews, because they did not wish to share personal information with people who they did not know. Further, the PRG members expressed that the openness and the straight-forward discussions in the group meetings provided a sense of security.

This matter was explained by one PRG member as follows:

A: Our common attitude is that all of us (PRG) have chosen for several years to be open and frank about our background. The fact that we have been actively engaged in user organizations is maybe not a criterion for our participation, but it has given us strength or necessary practice to dare verbalize our experiences. We don’t feel shame anymore about what we did wrong. We have developed further.

Their sense of security was strengthened through meeting regularly over time and thus getting to know the PI and the others in the group. This sense of security likely made it even more possible for the PRG members to open up about personal experiences (e.g., experiences of being deprived of child care responsibilities), which were important when reflecting on the research process.

The two group members who were recruited first were, to some degree, colleagues. The two who were recruited subsequently were each familiar with one of the two members recruited first, but did not know each other. A major focus during the group discussions concerned the content of the interviews and the search for recovery themes. The latter part of the group discussions opened up for more reflections on participation in the PRG and provided space for exploring the personal experiences of the PRG members, with respect to both the research process and the topics of SUDs and SUD recovery.

### The PI’s reflections of the research process

The PI had neither personal experience of having had a substance use disorder nor previous experience conducting or participating in collaborative research. The motivation for initiating the current project related, in part, to the steadily increasing demands of the research funding agencies in Norway that user groups be engaged when planning and conducting research on topics of direct relevance to them. In addition, a colleague at the Norwegian National Advisory Unit on Concurrent Substance Abuse and Mental Health Disorders had initiated a participatory research project one year prior [30], which became an inspiration for the current project. Further, the fact that MB had completed a course in collaborative research methods and was a long-term colleague also paved the way for undertaking a collaborative research project together.

When comparing qualitative collaborative research that includes peer researchers with traditional research approaches that do not, our experiences suggest that there are reasons to think that findings may vary between the two. In our instance, the PRG rephrased several of the interview questions that the PI had initially drafted, and the PRG interpreted some of the data and findings differently than the PI would have on his own. For instance, the PRG members discussed, on several occasions, what they perceived as the characteristics of successful SUD treatment and the topic of how, in particular, positive relationships can facilitate SUD recovery. These discussions emerged during the thematic development phase of the analysis and were based on experiences from their own SUD recovery. These topics likely received more emphasis in our analysis, and thus subsequently in our dissemination of findings, than they would have if this project had been conducted without collaborating with the PRG.

Engaging peer researchers in every aspect of the project was experienced as both challenging and rewarding. First and foremost, the PRG members had first-hand experiences with the study phenomena and could, to a certain extent, identify with the research participants on these grounds. Thus, it was possible to explore in-depth the meanings that the research participants expressed in the interviews, from this unique vantage point and the insider perspective that it offered. Further, the PI was challenged by the PRG to question rather than take for granted established knowledge concerning SUDs and recovery and to reflect consciously on the decision-making process. The PI was educated as a health worker and researcher and had thus predetermined the premises for both the recruitment of the research participants and the analytical method. Anyhow, the PRG members had strong opinions about how to collect the data, which questions to ask, how to phrase the questions, and what matters were most important during the analytical phase.

The PI expressed the following in the last meeting, when discussing experiences of the research process:

I have received feedback from the group that has changed my way of thinking in many ways of doing research with humans. This concerned mainly recognition of the diversity of SUD recovery trajectories and how my own preunderstanding can influence research activities. To actively collaborate with a user group makes the research project more time consuming and costly, but also provides a broader perspective and has been more of a social activity compared to traditional research.

The main challenge was to comply with the time schedule of the project. Considerable time was spent planning, scheduling, and preparing for the meetings with the PRG, as well as while disseminating information to and among the group members in between meetings. Likewise, it was necessary to apply for extra project funding to provide proper payment for the PRG members. On the other hand, the PI experienced the research process as less of an isolated working process, when compared to traditional research, and enjoying having a collaborative network with whom to exchange ideas and receive and provide social and professional support during the research process. Traditional research can be seen as a “vulnerable” process, in the sense that researchers often do not present the work before it is completed. By contrast, doing collaborative research entails a more transparent process of working alongside others, and facing questions and comments consecutively. What drove the project forward was a combination of professional and social engagement (e.g., meeting in the group, writing articles, and traveling abroad together).

## Discussion

The findings from this study suggest that the most important contributions of the PRG related to the development of the interview guide, preunderstanding among the PRG members, and the discussion of alternative forms of data collection. Additionally, the most notable of the PRG’s experiences of the research process related to the group aspect of participation, the value of predictable routines and clear expectations, and opening up about private matters. The PI’s experiences of the research process concerned being enriched by alternative ways of asking questions and interpreting findings, and participating in an interactive arena for reciprocal social and professional support.

Few previous studies have involved individuals with SUD experiences as research collaborators throughout the entire research process. A systematic review study identified 25 studies focusing on community-based research, ethical issues, and drug use. This review identified participant compensation, peer recruitment and representation, and capacity building as among the most important matters of ethical importance in collaborative research [10]. Only a minority of the identified studies in this review included peer researchers in recovery. In contrast, all PRG members in the current study were in stable recovery. There are, nevertheless, some similarities between the matters identified as important in those studies and those that have emerged as important in ours, such as proper compensation for PRG participants, as well as the possibility for capacity building and maybe even therapeutic gain. Similar findings are reported in studies utilizing a collaborative approach in the mental health field [31, 32]. In contrast, some studies have reported negative experiences with having engaged persons who actively use substances as co-researchers, such as discomfort with the research process, as well as confidentiality and safety concerns [33], and fears of exploitation and objectification [34].

Previous studies have identified a number of positive aspects of engaging people with lived experiences with SUDs, particularly with respect to recruitment and data collection [35, 36]. The current study entailed collaboration in study preparation, initial data analysis, and write-up, although the study design had been decided upon in advance and the study participants were recruited by the PI. Methodological studies of collaborative research in the field of SUD are sparse, and researchers wishing to conduct such research find themselves lacking tools to guide the process. By including peer researchers with SUD experience as equally valued partners in the data analysis, as in the current study, it is possible to achieve an in-depth understanding of the studied phenomena from the unique vantage point and insider perspective that this facilitates.

When interviewing individuals about potentially sensitive, stigmatized, and private lived experiences, the questions asked and manner of asking are of great concern. The concerns raised by the PRG members in the current study highlighted how an academic orientation can be a barrier to eliciting relevant information from study participants. This knowledge among the peer researchers was utilized to revise the initial interview guide and to adapt it at one point underway. However, the quality of the interview data, and possibly also the validity of findings, could have been potentially enhanced if the interview guide had been reviewed in all meetings and adapted continuously to account for emergent findings underway. The comparison of analytical findings between both each individual interview and between each set of four to five interviews can be seen as a validation process that likely strengthened the ongoing analysis throughout the research process.

Challenges of descriptive versus interpretive practices in analyzing qualitative data were prominent in the current study. The peer researchers found it challenging to distance themselves from the data and to “bracket” their lived experiences with SUD recovery when analyzing the transcribed interviews at a descriptive and thematic level. They may have experienced this expectation that they “bracket” their own experiences during the analysis process as a contradiction, considering that they were recruited because of their experiences with substance use and because these lived experiences were directly engaged when revising the interview guide and during discussions regarding the research process. It seemed that formal training in research collaboration was an advantage when facing this challenge and that working in a group of peers and alongside the PI, all of whom provided one another with feedback about these matters and challenged one another to examine the role of individual subjectivities and preunderstandings, was as well. The PI in this study had also gained knowledge about collaborative research between professional and peer researchers through discussions with researchers in comparable collaborative projects that had utilized similar methodologies. The research process may, however, be further enhanced if researchers receive formal training in collaborating with peer researchers in general, and within the SUD field in particular.

Findings from the current study indicate that establishing a structured process and a stable research environment was important for the PRG members. This structural element may be even more important when the participating peer researchers lack formal research training and education. Similarly, participating in a group with several peer researchers with a diversity of lived experiences regarding main substance(s) used, treatment, and recovery was seen as important for ensuring that multiple views were taken into account when analyzing the data. At the same time, when the PRG and the PI easily reached consensus about emerging themes, it was experienced by the peer researchers as indicative of valid findings and analysis, which, in turn, made it meaningful to take part in the research process.

Three out of four members of the PRG had no previous experience with research. Thus, it was not surprising that the discussion about the research process became more thorough and nuanced during the latter part of the project, as the PRG members gradually became more familiar with each other, the PI, the working tasks, and the steps in the data analyses. Thus, both the PI and the PRG members experienced more openness and engagement during the group discussions in the latter part of the project than in the beginning. At the same time, it was challenging to engage the entire PRG in the last group discussion, which occurred 3 years after the initiation of the process; only two PRG members participated in that meeting.

### Lessons learned

When establishing a PRG for the study of recovery processes, it can be advantageous to include several peer researchers with diverse substance use, treatment, and recovery experiences. If possible, at least one peer researcher with formal training or previous qualitative research experience should be included. Our experiences suggest that this person can, as an “insider” with respect to both research and substance use, help to recognize and bridge some of the gaps that might otherwise emerge and persist between the researchers without SUD backgrounds and the peer consultants without research experience.

In addition, the total time frame, schedule, work load, and payment should be presented when recruiting peer researchers. Sufficient funding should be secured in advance, so as to ensure proper payment during the entire project. Further, in each meeting during the process, it is important to discuss methodological issues and ensure that the workloads are suitable for all participants. Validity in the analytical process can be improved by engaging and drawing upon the lived experiences of the PRG members, with respect to both the thematic coding and analysis process itself and with respect to the adaptation of the interview guide for subsequent interviews. It is important to discuss, together with the PRG members and at the outset of the project, how to handle potential situations in which PRG members experience relapse or have difficulties performing their tasks.

The PI and PRG members may consider writing and publishing a popular scientific article in a journal, or publishing the findings of the research project elsewhere and in a lay language, so that they are accessible for study participants and communities with substance use experience.

Also, the PI should be trained in collaborating with peer researchers and/or should be part of a research environment in which it is possible to discuss methodological challenges with other researchers. Finally, the research process should be evaluated upon completion, by both the PI and the PRG. This should be done for the purpose of reflecting upon the ways in which the research process may have been improved and to consider opportunities for sharing this experiential learning and knowledge with local, national, and international SUD research environments.

## Data Availability

Not applicable.

## Abbreviations

SUD: Substance use disorder
OMT: Opioid maintenance treatment
COMORB study: The Comorbidity study: Substance Dependence and Co-occurrent Mental and Somatic Disorders
PRG: Peer research group
PI: Principal investigator
